# Public–private partnership alternative for a national pharmacare program in Canada

**DOI:** 10.1186/s40545-023-00526-3

**Published:** 2023-02-06

**Authors:** Eric Nauenberg, Emre Yurga

**Affiliations:** 1 Institute for Health Policy, Management and Evaluation, University of Toronto, Toronto, Canada; 2 Canadian Centre for Health Economics, University of Toronto, Toronto, Canada; 3 Toronto Health Economics and Technology Assessment Collaborative, University of Toronto, Toronto, Canada

**Keywords:** Pharmacare, Game theory, Free-ridership, Financing, Public–private partnerships

## Abstract

**Background:**

Recently, the government and an opposition party cut a deal that involved a promise to consider implementing a single-payer pharmacare scheme in Canada in exchange for supporting the current minority government. There have been political headwinds from the private extended health insurance industry, the provinces of Ontario and Quebec, as well as the pharmaceutical industry. We suggest a new multiple-payer of mixed-resort framework that achieves both the goal of universal coverage and preserves the private extended health insurance industry through a scheme based on the current coordination of benefits between private payers in this sector.

**Methods:**

We employ game theory to better understand the dynamics within a market that involves multiple payers. In particular, we use the game of Collective Action to help illustrate the problems of free-ridership.

**Results:**

An analysis of the dynamics of this market suggests that ex–ante agreements need to be struck between all payers in a multi-payer marketplace to achieve both stability and sustainability of such a framework.

**Conclusion:**

We show that universal coverage is still possible while leveraging the existing system of private extended health insurance so long as a well-established system for coordinating benefits between public and private payers is established. A stable public/private partnership can achieve universal coverage so long as a system for coordinating benefits is instituted. The proposed alternative will achieve the same goals, but maintain a niche for the private sector thereby maintaining therapeutic variety in the marketplace.

## Background

The current governing party in Canada—The Liberal Party of Canada—has recently entered into an agreement with one of the opposition parties to support the government in all confidence votes for the remainder of the mandate in exchange for action on a number of measures including the introduction of a national publicly funded pharmacare program with legislation slated for 2023 [1]. There have been some political headwinds from the insurance industry against implementation that openly expresses concern over supplanting their well-established territory in covering prescription drugs for a large portion of the population. Not only is this a major part of their business, there is a remaining question whether the pharmaceutical niche potentially supports the viability of the private insurance industry to offer other major areas of coverage—i.e., dental and vision not included in the pharmacare proposals [2]. As stated in an official position statement by the Canadian Life and Health Insurance Association—the trade association representing private extended health insurers—there exists a desire to maintain a vibrant private market under national pharmacare.

“We believe there is an achievable and affordable path forward that ensures all Canadians are able to access to the medications they need without putting at risk what’s working today…in our view, government plans and insurer-based plans should cooperate to negotiate lower drug prices for all Canadians. We also support a minimum standard list of drugs that all plans, whether public or private, should meet or exceed so there is more consistency across Canada and that existing gaps in coverage are closed” [3].

Varying levels of opposition to a single-payer pharmacare program have also been expressed by the Canadian Pharmacists Association, the pharmaceutical industry, as well as the Provinces of Ontario and Quebec [4–6]. As a result, it may be difficult to overcome stakeholder opposition to a national pharmacare system without incorporating elements of the current private marketplace. Steps forward towards universal coverage may therefore need to take compromise positions between what structurally currently exists and the proposed single-payer option. Ultimately, opposition stakeholders may prevail; as a result, producing a sufficient block of support may require a universal coverage scheme that conflates the goal of universal coverage with that of preserving private coverage for prescription drugs. Further, this may suggest some level of preference for a coordinated multi-payer system with the public system providing coverage in instances when no other coverage is available and at times possibly supplementing the coverage already provided by the private sector. While such a system may cost more than a single-payer system, it may also ensure supply of a greater assortment of prescription medications in the Canadian marketplace than if a single-payer system were to be able to negotiate bulk-purchasing agreements as a sole purchaser [7].

One of the problems with such a multi-payer system is creating stability amongst the payers over time so that there is a dynamic that discourages each payer from eventually shirking or pulling back from their financial position within the marketplace.^1^ In a system where multiple payers are responsible each for a portion of a claim, the tendency is to try to offload the financial responsibility for paying on other payers. Without adequate ex-ante provisions, a system that now includes a public payer sharing the financial burden of paying for prescription drugs may default eventually to a singlepublic-payer system regardless of whether the government is positioned as a primary payer (aka, payer of first-resort), a secondary payer or a payer of last-resort.

One of the large questions is the role that a government payer should assume when multiple sources of coverage are available to pay for a particular individual’s prescription drug bill. In the current paradigm in this policy space, non-universal public plans at the provincial level usually adopt the position of payer of first-resort limiting their ability to adapt to marketplace realities—an adaption recently made by the OHIP + program for those under age 25. This adjustment has resulted in large decreases in program outlays although whether access has decreased is yet to be determined; therefore, it is unclear whether a payer of first-resort strategy is optimal [8]. If a multi-payer national pharmacare option were to be ultimately chosen, this paper argues that the current system of coordinated coverage currently in place between private payers be extended to include a national public payer. This system involves ex-ante coordinating contractual agreements between the different payers (aka, payer of mixed-resort strategy). This will ultimately ensure that both market stability and the current breadth of therapeutic choice are sustained over the long-run.

### Theory and current market structure

Game theory helps to illustrate the dynamics between payers in an uncoordinated marketplace. The question arises whether government should be a first-payer, a secondary payer, or a payer of last resort when multiple sources of coverage are available to pay for a particular individual’s prescription drug bill.^2^ In the current system for prescription drug coverage, the public and private payers largely co-exist serving different niches of society. Currently, less than 60% of Canadians report having some level of private drug coverage [9]. Further, only between 5 and 10% of those with private coverage have multiple sources of coverage; hence, the impetus for coordination is currently not as strong as it would be if this 60% were also offered a public plan [10]. The current patchwork of coverage for prescription drugs is exemplified by Ontario which has universal public drug coverage with cost-sharing provisions for those over the age of 65, those with exceedingly high drug expenditures (i.e., those with drug expenditures that exceed approximately 3 to 4% of their income), those on social assistance, those in long-term care facilities, those receiving home care services, and those under the age of 25 who are not covered by a private plan. Some of these publicly covered populations can ostensibly also have private coverage in addition to their public coverage such as those over the age of 65 and those with exceedingly high prescription drug expenditures. While ensuring universal coverage is the ultimate goal, there is currently no integrated framework for cost-sharing between public and private payers but rather a fragmented system of varying sources of coverage for different populations. One possible solution is a payer of mixed-resort strategy in which a new national payer for prescription drugs could be inserted into a system already employed by private insurers to coordinate benefits (e.g., each spouse may have a plan through their respective employers which also includes coverage for dependents).^3^

Game theory involving iterated games may help to illustrate the strategies used by payers/employers^4^ that point out the need for such ex-ante coordination schemes to enhance market stability [11]. These games can either be two-player (e.g., a public payer and a single private payer/employer) or multi-player (three or more payer/employer) repeated games (e.g., a public payer and two or more private payers). In such games, each payer has a choice of staking out a position as a payer of firstresort, a second payer, or possibly even a payer of last-resort hoping to free-ride on the larger contribution of the other payers; however, each payer is willing to bear the position of payer of first-resort if no other payer is willing to do so. For the various scenarios faced, the game can be illustrated as either a two-payer or three-payer repeated game with anticipated—but possibly not realized—payouts (i.e., negative payoffs).

In a two-player scenario in Table 1, the payouts (75₵ on the dollar for payer of first-resort and 25₵ on the dollar for last-resort payers) suggest that both sources of coverage will gravitate toward the southeast cell of the matrix where both attempt to be payers of last-resort. Likewise, in the three-player scenario of Table 2, all payers will gravitate to a payer of last resort strategy ending up in equilibrium—the lower table in the southeast cell. This pure strategy involves minimizing the financial obligation on any particular claim and might eventually even lead to exit from the market altogether. In essence, following a strategy of last-resort is an attempt to free-ride on others who are positioned as a payer of first-resort. In the end, the laissez-faire equilibrium obtained may not be *pareto optimal* as not all costs and benefits associated with each position are fully internalized within the market.

**Table 1:**
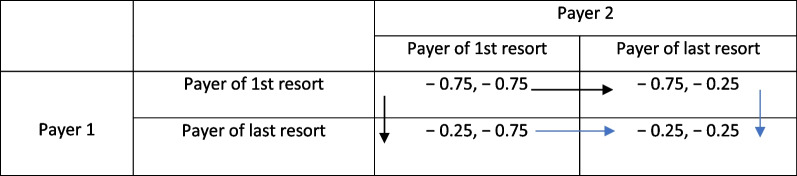
Two-payer repeated game

**Table 2:**
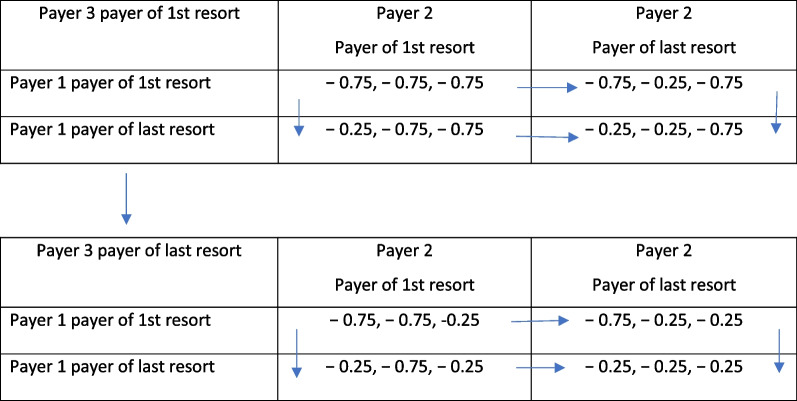
Three-payer repeated game

This conundrum can be understood as a form of the Collective Action Problem [12–14]. This version of the game consists of a failure to attain the most socially preferred outcome as a group because each payer serves themselves individually with a strictly dominant strategy. In essence, each payer holds back with a result that the group as a whole fails to attain an optimal result. Given a social preference for a multi-payer system, it would behoove all payers to cooperate by coordinating payments amongst themselves with shared financial burdens for providing coverage. From the point-of-view of game theory, obtaining such a result would involve including an additional player in the background who randomly assigns payer positions which could be considered “..moves by nature.” or rolling the dice [15]. In this particular case, this additional player could be represented by an ex-ante contractual arrangement that results in equitable sharing between positions of first-, second-, and last-resort through some type of sorting mechanism.

### Solution to free-rider problem under a coordinated benefits scheme

The solution to market stability requires examining the market at the level of an individual claim. A brochure put out by the Canadian Life and Health Insurance Association (CLHIA) helps to elucidate the current coordination of benefits between private payers based on the date of birth of each of the policy holders [16]. The policy-holder with the earlier birthday in the calendar year is established as the policy holder having the primary insurance policy to pay a prescription claim and the other policy-holder is considered to have the secondary insurer (which would be responsible for covering copays and deductibles up to the limits of coverage established by the secondary insurer). A proposed universal pharmacare program could be incorporated into this scheme so that the responsibility of paying for prescription drugs is shared between existing private sources of financing and the universal program to produce an outcome that would not have occurred without such an agreement. Such a scheme would achieve the goal of universal coverage without displacing the private insurance industry.

Considering this coordination of payments strategy, there are three types of individuals that could face a new publicly financed prescription drug benefit.

#### Case A: a person has no private coverage

In this instance, the publicly financed plan would be the sole source of coverage for prescription drugs covering up to the limits established within the program.

#### Case B: a person has one source of private coverage

In this instance, a person now will have two sources of coverage which can be coordinated much like a person currently who is doubly covered under spousal/family arrangements. Under this scheme, a person who has a birthday in the last half of the year (July to December) would be primarily covered by the publicly financed system and secondarily covered by their private plan. The opposite would be true for those with birth-dates during the first half of the year (January to June). This should be inherently stable with neither source of insurance coverage experiencing an unstable equilibrium over time as the burden would average out assuming a uniform distribution of birth-dates amongst those in the population.

#### Case C: a person has two sources of private coverage (spousal/familial arrangements)

With three sources of coverage, the proposal is to insert the public payer into this scheme as follows when there are three parties (public payer and two private payers):

1. If the birthdays of the two policy holders both fall in the months from May to December (the last two-thirds of the year), then the publicly financed system would assume primary responsibility for coverage and the private insurers would assume responsibility for secondary coverage (i.e., paying for copays and deductibles under the publicly financed system with the policy-holder with the earlier birthday assuming secondary coverage and the other policy-holder assuming tertiary coverage.
2. If the birthdays of the two policy holders both fall in the months from January to August (the first two-thirds of the year), then the publicly financed system would assume tertiary responsibility for coverage (i.e., paying that portion of the bill not covered by the other two private payers) and the private insurers would assume primary and secondary coverage (i.e., the policy holder with the earlier birthday would have the policy established as the primary source of coverage and the person with the latter birth date during the calendar year having the policy assuming the secondary payer role)
3. If the birthdates of the two individuals fall within the first third and last third of the year, then the policy-holder with the earlier birthdate would have a policy established as the primary payer, the publicly financed system would assume the secondary payer role, and the other private policy would assume the tertiary coverage.

For this scheme to operate continuously into the future, there will need to be some sort of mandate like that currently in Quebec whereby any firm providing health benefits will need to provide a minimum level of prescription drug benefits equivalent to the publicly financed prescription drug program (RAMQ), and people eligible for coverage under a private group extended health insurance plan must join and pay the premiums for such a group [17]. The end result of this scheme is a payer of mixed-resort strategy (*p* = 0.5 in a two-payer framework and 0.33 in a situation with three payers) in which the primary responsibility for primary payment will be uniformly distributed amongst the payers over the population of prescription drug claims submitted.

## Discussion

The problem of free-ridership occurs more frequently as the number of people with multiple sources of coverage increases with the rollout of a new universal public payer. Therefore, some type of contractual arrangement—or at least a *modus vivendi* by which all payers agree to abide—is necessary to ensure that all participating payers share the financial burden of covering mutual beneficiaries; otherwise, the equilibria predicted by game theory would produce results that are less than optimal. The payer of mixed-resort strategy proposed achieves this optimality and stability over the long run by alternating the position of payers—first-, second-, or third-resort—based on when the birthdays of policy holders fall during the calendar year. Further, by maintaining private insurance coverage, there remains the potential for a market for those prescription medications that do not find there way on to the public formulary thereby encouraging more therapeutic variety than otherwise might be available. While one of the key attractions of a single-payer option is the potential for bulk-purchasing discounts, such a scheme could limit therapeutic variety and worsen health outcomes by both establishing monopoly suppliers in exchange for negotiated price discounts and reduce the rate of introduction of new prescription drugs [7, 18–20]. The alternative proposed balances the need to maintain therapeutic variety with the attractions of bulk-purchasing by maintaining a market for pharmaceutical products not contained within the public formulary.

The current scheme by which all private insurers currently coordinate benefits could be adapted to include a public payer so long as prices are renegotiated for those prescription drugs that appear on the public formulary. To further strengthen this arrangement with performance metrics, it might be valuable to establish a report-back mechanism through which data are reported publicly regarding the percentage that each party—i.e., each player in game-theoretic terms—paid of each province’s total prescription drug bill and the proportion that each assumed as payer of first-, second- and/or last-resort in prescription drug transactions. In the end, the expectation is that the alternative policy proposed would lessen taxpayers’ burden of supporting universal prescription drug coverage and maintain therapeutic variety in the marketplace [7, 20]. Possibly even more importantly, the launch of new innovative medicines and less expensive generic drugs may be delayed; this may be the result of market-wide bulk-purchasing agreements that can also lead to decrements in health outcomes leading to other non-pharmaceutical health care expenditures and even avoidable downstream prescription costs. Such is still hotly debated [21, 22].

Finally, a coordinated multi-payer system may indeed be less cost-efficient at adjudicating claims than are public payers. Law, Kratzer and Dhalla [23] find that the percentage of premiums collected that go into paying out prescription drug benefits has decreased substantially in the private sector since 1990 leading to a gap between premiums collected and benefits paid of $6.8 billion as of 2011 [23]. The cost of adjudicating claims is much less in publicly funded programs suggesting that a multi-payer system would not be overall cost-efficient compared to a single-payer system; yet, multi-payer systems may still be the preference of a majority of stakeholders in this niche as *pareto optimality* is not necessarily determinative of the ultimate policy direction taken.

At this point in time, the private and public sectors also pay at different rates for the same drugs and have different formularies that are available for coverage. While this scheme would work even if the formulary lists differed, there may be a need to iron out differences in how drugs are priced according to who now pays the bill (e.g., current public plans remunerate at a lower base price than do the private insurers). For this scheme to establish a stable equilibrium over time, these differences in price would have to be adjusted such that prices are generally similar across payers.

## Conclusion

The penetration of private prescription drug insurance coverage and current relationships between public and private coverage vary across the provinces and territories dictating how a new national pharmacare program could be rolled out in different provinces and territories. While Ontario has fairly high penetration of such coverage, Newfoundland and Labrador has very low penetration and currently relies heavily on public sources of coverage for those who qualify for such assistance. Quebec has a well-established relationship between RAMQ and private insurers that leads to de facto province-wide pharmacare program in that province. BC has a Fair Pharmacare program that provides essentially catastrophic public coverage for prescription drugs for everyone. Thus, the current situation in each province will likely determine how a new federal prescription drugs program will liaise with current systems in each province. This paper suggests maintaining the current penetration of private prescription drug insurance in the marketplace coordinating payments with a national public payer to maintain market stability while sustaining therapeutic variety.

## Footnotes

1 Evan a public payer can pull back on their position—witness Ontario’s OHIP + which was introduced in 2018 as universal first-payer pharmaceutical coverage for those under age 25 only to be pulled back in the Spring of 2019 to exclude those covered under private extended health care plans.

2 There are instances when a person only has publicly provided coverage for prescription drugs; therefore, there is no need for coordination in such instances. In Ontario, these include people over age 65 without private insurance coverage for prescription drugs and those on social assistance as well as those under the age of 25 without parental private insurance coverage for prescription drugs.

3 We acknowledge that there are people in the population for which their only source of coverage is a public payer. In this case, the proposed scheme obviates the need for coordination amongst payers.

4 The vast majority of private extended insurance products in Canada are classified as administrative services only (ASO) in which the employer bears the financial risk of covering their employers prescription drug expenditures; thus, the ultimate payer is most often the employer.
